# Sulfonamide-Based Azaheterocyclic Schiff Base Derivatives as Potential Carbonic Anhydrase Inhibitors: Synthesis, Cytotoxicity, and Enzyme Inhibitory Kinetics

**DOI:** 10.1155/2020/8104107

**Published:** 2020-02-20

**Authors:** Mujahid Abas, Hummera Rafique, Shazia Shamas, Sadia Roshan, Zaman Ashraf, Zafar Iqbal, Hussain Raza, Mubashir Hassan, Khurram Afzal, Albert A. Rizvanov, Muhammad Hassham Hassan Bin Asad

**Affiliations:** ^1^Department of Chemistry, Allama Iqbal Open University, Islamabad 44000, Pakistan; ^2^Department of Chemistry, University of Gujrat, Gujrat-50700, Pakistan; ^3^Department of Zoology, University of Gujrat, Gujrat-50700, Pakistan; ^4^Department of Biological Sciences, College of Natural Sciences, Kongju National University, Gongju 314-701, Republic of Korea; ^5^Institute of Molecular Biology and Biotechnology, The University of Lahore, Pakistan; ^6^Institute of Food Sciences, Bahauddin Zakria University, Multan 60800, Pakistan; ^7^Institute of Fundamental Medicine and Biology, Department of Genetics, Kazan Federal University, 420008 Kazan, Russia; ^8^Department of Pharmacy, COMSATS University Islamabad, Abbottabad Campus 22060, Pakistan

## Abstract

A series of sulfonamide-bearing azaheterocyclic Schiff base derivatives **3(a-j)** were synthesized as carbonic anhydrase inhibitors. The substituted benzene sulfonyl chlorides **1(a-d)** were reacted with N_2_H_4_ to get aromatic sulfonyl hydrazides **2(a-d)**. The intermediate hydrazides **2(a-d)** were treated with substituted aldehydes to afford azaheterocyclic sulfonamide Schiff bases **3(a-j)**. The spectral data of synthesized compounds confirmed the formation of the final products. The inhibitory effects of **3(a-j)** on carbonic anhydrase activity were determined, and it was found that derivative **3c** exhibited the most potent activity with IC_50_0.84 ± 0.12 *μ*M among all other derivatives and is also more active than standard acetazolamide (IC_50_0.91 ± 0.12). The enzyme inhibitory kinetics results determined by Lineweaver-Burk plots revealed that compound **3c** inhibits the enzyme by noncompetitive mode of inhibition with *K*_i_ value 8.6 *μ*M. The molecular docking investigations of the synthesized analogues **3(a-j)** were evaluated which assured that synthesized compounds bind well inside the active binding site of the target enzyme. Cytotoxicity on human keratinocyte (HaCaT) and MCF-7 cell lines was performed, and it was found that most of the synthesized analogues were nontoxic on these cell lines and the toxic effects follow the dose-dependent manner. Based on our investigations, it was suggested that analogue **3c** may serve as core structure to project carbonic anhydrase inhibitors with greater potency.

## 1. Introduction

Carbonic anhydrase (CA) (EC#: 4.2.1.1) is a metal group containing enzymes that hydrate CO_2_ to bicarbonate ions and vice versa [1]. Various CA isoforms are classified by their structures. Based upon their hosts, they are further categorized as *α*-CAs and are observed primarily in algae, plants, and vertebrates; *β*-CAs are expressed in bacteria, fungi, and algae; *γ*-carbonic anhydrase appears in bacteria and archaea [2]. Two minor isoforms of CA, *δ* and *ζ*, are primarily expressed in marine diatoms and *η* in protozoans, respectively [3]. For their catalytic domains, crystal structures of all isoforms were studied and submitted to the Protein Data Bank (PDB) [4].

The *α*-CAs' catalytic area consists of 10 stranded *β* sheets and seven *α* helices around the surface [5]. The CA catalytic active site is designed conically in which a zinc atom exists on the base that is harmonized with three histidine moieties (His94, His96, and His119) and a hydroxide ion/water [6]. Most structural information in the field of CA emphasize on the universally expressed human CA II, as it is very abundant and effective and is an isoform which can be easily crystallized [7]. The catalytic active site residues of CA II are alienated into hydrophobic and hydrophilic halves, through which the carbon dioxide substrate enters the active site [8]. The mode of enzymatic activity of the carbonic anhydrase consists of two steps [9]. In the hydration procedure, as an initial step, a nucleophilic zinc-bound hydroxyl assaults carbon dioxide and give rise to a zinc-bound bicarbonate particle. The bicarbonate is pitifully bound to the zinc atom and is consequently in this manner replaced by a water molecule. The second step of this enzymatic response is the recovery of the zinc-bound hydroxyl, which happens by means of proton transfer [10].

Using a proton donor/acceptor His64 residue and well-ordered network of water molecules in the active site of CA II, the transfer of proton is enabled [11]. Depending on the protonation state, His64 represents two conformations: a conformation in which His64 appears inside the active site to receive a proton called “in,” while a conformation called “out” where the proton containing His64 residue faces away from active place of the active site to allow transferring of proton toward the bulk solvent [12]. A wide variety of activities have been associated with CAs, and a lot of pharmacological functions have been found to have close relationship with activation or inhibition of CA [13]. Antiglaucoma, antitumor, antiobesity, and anticonvulsant drugs are examples of such pharmacological drugs [14]. Those inhibitors which affect hCA II, IV, XII, and XIV serve as diuretic medications [15]. While inhibitors which can affect hCA II, IV, and XII act as antiglaucoma drugs. Inhibitors affecting hCA II, VII, and XIV perform antiepileptic activities [16]. Presently, hCA IX and XII have shown their role as an investigative source to imaging and curing the hypoxic tumors by using a sulfonamide (SLC-0111) in Phase I/II clinical trials to treat growing metastatic breast cancers [17]. CA inhibition has been widely explored using aromatic heterocycles containing Schiff bases [18]. Aromatic heterocycles with Schiff bases are very valuable for having dual character in ditopic interactions in the enzymatic active sites, achievable during the event of dimeric carbonic anhydrases [19]. Multitopic carbonic anhydrase inhibitors of the sulfonamides have been investigated to hold an improved inhibition for several isoforms of carbonic anhydrase which comprise dimeric, trimeric, or tetrameric associations [20, 21].

Based upon the importance of sulfonamide-based aromatic heterocycles, the present research is designed to synthesize novel substituted azaheterocyclic sulfonamide Schiff bases as carbonic anhydrase inhibitors. The computational studies were also carried out to check the binding affinity of the synthesized compounds against target protein PDBID 1V9E. The in vitro carbonic anhydrase inhibitory activity was determined, and kinetic mechanism of the most active compound was also investigated. The cytotoxicity of the synthesized compounds against human keratinocyte (HaCaT) and MCF-7 cell lines was also studied.

## 2. Material and Methods

### 2.1. Chemicals and Instruments

All the chemicals and carbonic anhydrase enzyme (EC#: 4.2.1.1) used have been obtained from Sigma-Aldrich. The FTIR studies have been performed using a Shimadzu FTIR e8400S spectrometer (Kyoto, Japan, y, cm^−1^). Results of ^1^H and ^13^C NMR spectral data (DMSO-d_6_) and (CDCl_3_) have been obtained from a Bruker (400 MHz) spectrometer. The chemical shift values (*d*) have been shown in ppm downfield using TMS (tetramethylsilane) as an internal standard. Purity of synthesized analogues has been determined by using TLC (thin layer chromatography) with ethyl acetate and petroleum ether as mobile phase. The complete procedure to synthesize these target derivatives has been shown in the scheme below.

### 2.2. Synthesis of Aromatic Sulphonyl Hydrazides **2(a-d)**

Aromatic sulphonyl chlorides **1(a-d)** (0.01 mol) in liquid state were stirred with hydrazine in a 100 mL flask for one hour. On completion, the mixture contents were allowed to mix with cold water. Precipitates of aromatic sulphonyl hydrazides **2(a-d)** formed were collected and dried which were characterized by their IR spectral data.

### 2.3. Synthesis of Azaheterocyclic Sulfonamide Schiff Bases **3(a-j)**

**Scheme 1:**
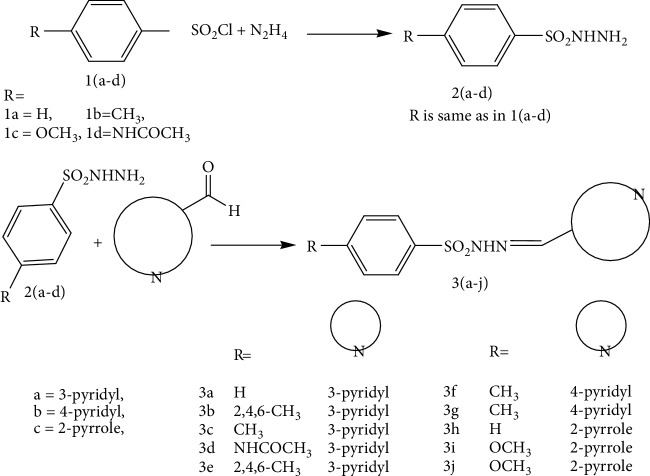
Synthesis of azaheterocyclic Schiff base sulfonamide derivatives **3(a-j)**

Aromatic sulphonyl hydrazides **2(a-d)** (10 mmol) and *p*-substituted benzaldehydes in equimolar ratio in 10 mL of dry ethanol were taken in a flask. Contents of the reaction mixture were refluxed for 12 hours, and reaction was monitored by TLC using (petroleum ether : ethyl acetate 3 : 1) as eluants. On completion of reaction, the reaction mixture was dropped in ice-cool water. Precipitates of Schiff bases **3(a-j)** that appeared were collected, dried, and crystallized using aqueous ethanol. By using the already-reported method [22], the title compounds **3(a-j)** were synthesized with certain modifications as shown in Scheme 1.

#### 2.3.1. (*E*)-N′-(pyridin-3-ylmethylene)benzenesulfonohydrazide (**3a**)

Solid; reaction time, 12 h; yield, 45%; melting point: 176–178°C; *R*_f_ 0.47 (ethyl acetate : pet. ether 1 : 3); FTIR *n*_max_ cm^1^: 3356 (NH), 3035 (sp^2^ CH), 1676 (CN), 1421 (C=C aromatic). HPLC result of product: Daicel CHIRALPAK® AD-H column; 30% ethyl acetate in pet. ether; 1.0 mL/min; retention time: 33.9 min; 99.3% pure; ^1^H NMR (CDCl_3_, *d* ppm, *J* Hz); 7.07 (*d*, *J* = 8.0, 2H, H-3′, H-4′), 7.01 (*d*, *J* = 8.0, 2H, H-2′, H-5′), 6.92 (s, 2H, H-3, H-5), 6.74 (s, 1H, CH), 3.74 (s, 1H, NH), 2.14 (s, 3H, CH_3_), 2.07 (s, 6H, CH_3_). ^13^C NMR (CDCl_3_, *d* ppm); 145.1 (CH), 144.4 (C-1), 142.9 (C-1′), 142.3 (C-2′, C-5′), 139.4 (C-4), 132.4 (C-3′, C-4′), 129.8 (C-3, C-5), 129.3 (C-2, C-6), 127.4 (C-3′, C-5′), 22.4 (CH_3_), 21.3 (CH_3_). LCMS (H+) calculated for C_12_H_11_N_3_O_2_S [M–H]^+^: 262.06, found: 262.30; anal. calculated (%): C, 55.16; H, 4.24; N, 16.08. Found: C, 55.01; H, 4.16; N, 16.10.

#### 2.3.2. (*E*)-2,4,6-trimethyl-N′-(pyridin-3-ylmethylene)benzenesulfonohydrazide (**3b**)

Solid; reaction time, 12 h; yield, 43%; melting point: 173–176°C; *R*_f_ 0.44 (ethyl acetate : pet. ether 1 : 3), FTIR *n*_max_ cm^1^: 3149 (NH), 3062 (sp^2^ CH), 1622 (CN), 1442 (C=C aromatic). HPLC result of product: Daicel CHIRALPAK® AD-H column; 30% ethyl acetate in pet. ether; 1.0 mL/min; retention time: 26.8 min; 97% pure; ^1^H NMR (CDCl_3_, *d* ppm, *J* Hz); 10.11 (s, 1H, CH), 8.89 (*d*, *J* = 8.0, 2H, H-2′, H-5′), 8.53 (*d*, *J* = 4.0, 2H, H-2, H-6), 7.82 (*d*, *J* = 4.0, 2H, H-3′, H-4′), 7.31 (*d*, *J* = 4.0, 2H, H-3, H-5), 4.51 (s, 1H, NH), 1.18 (s, 3H, -CH_3_), 2.07 (s, 6H, -CH_3_). ^13^C NMR (CDCl_3_, *d* ppm); 193.8 (CH), 151.5 (C-1), 149.9 (C-2, C-6), 148.9 (C-3, C-5), 148.2 (C-1′), 122.5 (C-4), 122.9 (C-2′, C-5′), 122.1 (C-3′, C-4′), 15.4 (CH_3_). LCMS (H+) calculated for C_15_H_17_N_3_O_2_S [M–H]^+^: 304.38, found: 304.19; anal. calculated (%): C, 59.38; H, 5.65; N, 13.85. Found: C, 59.29; H, 5.46; N, 13.60.

#### 2.3.3. (*E*)-4-methyl-N′-(pyridin-3-ylmethylene)benzenesulfonohydrazide (**3c**)

Solid; reaction time, 12 h; yield, 47%; melting point: 168–171°C; *R*_f_ 0.43 (ethyl acetate : pet. ether 1 : 3), FTIR *n*_max_ cm^1^: 3379 (NH), 2972 (sp^2^ CH), 1612 (CN), 1427 (C=C aromatic). HPLC result of product: Daicel CHIRALPAK® AD-H column; 30% ethyl acetate in pet. ether; 1.0 mL/min; retention time: 25.4 min; 99.5% pure; ^1^H NMR (DMSO-d_6_, *d* ppm, *J* Hz); 11.24 (s, 1H, CH), 10.92 (s, 1H, NH), 7.77 (*d*, *J* = 8.0, 2H, H-2, H-6), 7.37 (*d*, *J* = 8.0, 1H, H-4′), 7.37 (*d*, *J* = 8.0, 2H, H-3, H-5), 6.85 (*d*, *J* = 8.0, 1H, H-2′), 6.35 (s, 1H, NH), 6.06 (*d*, *J* = 8.0, 4.0, 1H, H-3), 2.36 (s, 3H, -CH_3_). ^13^C NMR (DMSO-d_6_, *d* ppm); 143.6 (CH), 140.9 (C-1), 136.7 (C-4′), 129.9 (C-2, C-6), 127.7 (C-3, C-5), 126.8 (C-3′), 122.7 (C-2′), 113.5 (C-1′), 109.5 (C-4), 21.9 (CH_3_). LCMS (H+) calculated for C_13_H_13_N_3_O_2_S [M–H]^+^: 275.02, found: 275.19; anal. calculated (%): C, 56.71; H, 4.76; N, 12.56. Found: C, 56.43; H, 4.86; N, 13.10.

#### 2.3.4. (*E*)-N-(4-((2-(pyridin-3-ylmethylene)hydrazinyl)sulfonyl)phenyl)acetamide (**3d**)

Solid; reaction time, 12 h; yield, 59%; melting point: 181–184°C; *R*_f_ 0.42 (ethyl acetate : pet. ether 1 : 3), FTIR *n*_max_ cm^1^: 3300 (NH), 3132 (sp^2^ CH), 1531 (CN), 1419 (C=C aromatic). HPLC result of product: Daicel CHIRALPAK® AD-H column; 30% ethyl acetate in pet. ether; 1.0 mL/min; retention time: 19.5 min; 100% pure; ^1^H NMR (CDCl_3_, *d* ppm, *J* Hz); 11.27 (s, 1H, CH), 11.01 (s, 1H, NH), 7.91 (*d*, *J* = 4.0, 2H, H-2, H-6), 7.75 (*d*, *J* = 4.0, 1H, H-4′), 7.57 (m, 3H, H-3, H-4, H-5), 6.85 (*d*, *J* = 8.0, 1H, H-2′), 6.36 (s, 1H, NH), 6.06 (*d*, *J* = 8.0, 4.0, 1H, H-3), 2.36. ^13^C NMR (CDCl_3_, *d* ppm); 162.9 (CH), 140.9 (C-1), 131.3 (C-4′), 129.9 (C-2, C-6), 126.9 (C-3′), 122.6 (C-2′), 114.6 (C-3, C-5), 113.3 (C-1′), 109.5 (C-4). LCMS (H+) calculated for C_14_H_14_N_4_O_3_S [M–H]^+^: 319.08, found: 319.35; anal. calculated (%): C, 52.82; H, 4.43; N, 17.60. Found: C, 52.79; H, 4.16; N, 17.40.

#### 2.3.5. (*E*)-2,4,6-trimethyl-N′-(pyridin-4-ylmethylene)benzenesulfonohydrazide (**3e**)

Solid; reaction time, 12 h; yield, 51%; melting point: 160–164°C; *R*_f_ 0.29 (ethyl acetate : pet. ether 1 : 3), FTIR *n*_max_ cm^1^: 3319 (NH), 3023 (sp^2^ CH), 1662 (CN), 1441 (C=C aromatic). HPLC result of product: Daicel CHIRALPAK® AD-H column; 30% ethyl acetate in pet. ether; 1.0 mL/min; retention time: 21.2 min; 99.5% pure; ^1^H NMR (CDCl_3_, *d* ppm, *J* Hz); 11.24 (s, 1H, CH), 10.92 (s, 1H, NH), 7.77 (*d*, *J* = 8.0, 2H, H-2, H-6), 7.37 (*d*, *J* = 8.0, 1H, H-4′), 7.37 (*d*, *J* = 8.0, 2H, H-3, H-5), 6.85 (*d*, *J* = 8.0, 1H, H-2′), 6.35 (s, 1H, NH), 6.06 (*d*, *J* = 8.0, 4.0, 1H, H-3), 2.36 (s, 3H, -CH_3_). ^13^C NMR (CDCl_3_, *d* ppm); 143.6 (CH), 140.9 (C-1), 136.7 (C-4′), 129.9 (C-2, C-6), 127.7 (C-3, C-5), 126.8 (C-3′), 122.7 (C-2′), 113.5 (C-1′), 109.5 (C-4), 21.9 (CH_3_). LCMS (H+) calculated for C_15_H_17_N_3_O_2_S [M–H]^+^: 304.11, found: 304.19; anal. calculated (%): C, 59.38; H, 5.65; N, 13.85. Found: C, 59.19; H, 5.65; N, 13.85.

#### 2.3.6. (*E*)-4-methyl-N′-(pyridin-4-ylmethylene)benzenesulfonohydrazide (**3f**)

Solid; reaction time, 12 h; yield, 63%; melting point: 148–152°C; *R*_f_ 0.36 (ethyl acetate : pet. ether 1 : 3), FTIR *n*_max_ cm^1^: 3298 (NH), 3035 (sp^2^ CH), 1598 (CN), 1453 (C=C aromatic). HPLC result of product: Daicel CHIRALPAK® AD-H column; 30% ethyl acetate in pet. ether; 1.0 mL/min; retention time: 30.1 min; 88% pure; ^1^H NMR (CDCl_3_, *d* ppm, *J* Hz); 11.26 (s, 1H, CH), 10.83 (s, 1H, NH), 7.83 (*d*, *J* = 8.0, 2H, H-2, H-6), 7.74 (*d*, *J* = 8.0, 1H, H-4′), 7.08 (*d*, *J* = 8.0, 2H, H-3, H-5), 6.84 (*d*, *J* = 8.0, 1H, H-2′), 6.34 (s, 1H, NH), 6.06 (*d*, *J* = 8.0, 4.0, 1H, H-3), 3.82 (s, 3H, -OCH_3_). ^13^C NMR (CDCl_3_, *d* ppm); 167.9 (CH), 140.9 (C-1), 131.3 (C-4′), 129.9 (C-2, C-6), 126.9 (C-3′), 122.6 (C-2′), 114.6 (C-3, C-5), 113.3 (C-1′), 109.5 (C-4), 56.0 (OCH_3_). LCMS (H+) calculated for C_13_H_13_N_3_O_2_S [M–H]^+^: 276.07, found: 276.33; anal. calculated (%): C, 56.71; H, 4.76; N, 15.26. Found: C, 56.69; H, 4.69; N, 15.10.

#### 2.3.7. (*E*)-4-methyl-N′-(pyridin-2-ylmethylene)benzenesulfonohydrazide (**3g**)

Solid; reaction time, 12 h; yield, 53%; melting point: 159–161°C; *R*_f_ 0.53 (ethyl acetate : pet. ether 1 : 3), FTIR *n*_max_ cm^1^: 3281 (NH), 3031 (sp^2^ CH), 1671 (CN), 1411 (C=C aromatic). HPLC result of product: Daicel CHIRALPAK® AD-H column; 30% ethyl acetate in pet. ether; 1.0 mL/min; retention time: 25.3 min; 97.9% pure; ^1^H NMR (CDCl_3_, *d* ppm, *J* Hz); 7.67 (s, 1H, CH), 7.39 (*d*, *J* = 8.0, 2H, H-2, H-6), 7.34 (*d*, *J* = 8.0, 2H, H-2′, H-5′), 7.30 (*d*, *J* = 8.0, 2H, H-3′, H-4′), 7.13 (m, 3H, H-3, H-4, H-5), 6.19 (s, 1H, NH), 2.11 (s, 1H, CH_3_). ^13^C NMR (CDCl_3_, *d* ppm); 149.1 (CH), 146.3 (C-1), 143.4 (C-1′), 141.5 (C-2′, C-5′), 139.2 (C-4), 129.2 (C-3′, C-4′), 126.7 (C-3, C-5), 126.1 (C-2, C-6), 125.2 (C-3′, C-5′), 21.3 (CH_3_). LCMS (H+) calculated for C_18_H_18_N_3_O_2_S_3_Cl [M–H]^+^: 276.07, found: 276.09; anal. calculated (%): C, 56.71; H, 4.76; N, 15.26. Found: C, 56.79; H, 4.56; N, 15.54.

#### 2.3.8. (*E*)-N′-((1H-pyrrol-2-yl)methylene)benzenesulfonohydrazide (**3h**)

Solid; reaction time, 12 h; yield, 58%; melting point: 156–158°C; *R*_f_ 0.37 (ethyl acetate : pet. ether 1 : 3), FTIR *n*_max_ cm^1^: 3296 (NH), 3088 (sp^2^ CH), 1611 (CN), 1475 (C=C aromatic). HPLC result of product: Daicel CHIRALPAK® AD-H column; 30% ethyl acetate in pet. ether; 1.0 mL/min; retention time: 20.4 min; 99.9% pure; ^1^H NMR (CDCl_3_, *d* ppm *J* Hz); 7.73 (s, 1H, CH), 7.47 (*d*, *J* = 8.0, 2H, H-2, H-6), 7.44 (*d*, *J* = 8.0, 2H, H-2′, H-5′), 7.40 (*d*, *J* = 8.0, 2H, H-3′, H-4′), 7.14 (*d*, *J* = 8.0, 2H, H-3, H-5), 6.31 (s, 1H, NH), 3.31 (s, 1H, OCH_3_). ^13^C NMR (CDCl_3_, *d* ppm); 151.1 (CH), 147.5 (C-1), 143.6 (C-1′), 142.6 (C-2′, C-5′), 140.6 (C-4), 133.3 (C-3′, C-4′), 128.7 (C-3, C-5), 126.2 (C-2, C-6), 125.3 (C-3′, C-5′), 51.4 (OCH_3_). LCMS (H+) calculated for C_11_H_11_N_3_O_2_S [M–H]^+^: 250.06, found: 250.01; anal. calculated (%): C, 53.00; H, 4.45; N, 16.86. Found: C, 53.09; H, 4.36; N, 16.70.

#### 2.3.9. (*E*)-N′-((1H-pyrrol-2-yl)methylene)-4-methylbenzenesulfonohydrazide (**3i**)

Solid; reaction time, 12 h; yield, 67%; melting point: 146–148°C; *R*_f_ 0.42 (ethyl acetate : pet. ether 1 : 3), FTIR *n*_max_ cm^1^: 3287 (NH), 3135 (sp^2^ CH), 1576 (CN), 1461 (C=C aromatic). HPLC result of product: Daicel CHIRALPAK® AD-H column; 30% ethyl acetate in pet. ether; 1.0 mL/min; retention time: 24.7 min; 99% pure; ^1^H NMR (CDCl_3_, *d* ppm, *J* Hz); 11.34 (s, 1H, CH), 11.23 (s, 1H, NH), 8.39 (*d*, *J* = 8.0, 2H, H-2, H-6), 8.15 (*d*, *J* = 8.0, 2H, H-3, H-5), 7.77 (*d*, *J* = 8.0, 1H, H-4′), 6.88 (*d*, *J* = 8.0, 1H, H-2′), 6.40 (s, 1H, NH), 6.08 (*d*, *J* = 4.0, 1H, H-3′). ^13^C NMR (CDCl_3_, *d* ppm); 175.1 (CH), 142.5 (C-1), 134.8 (C-4′), 129.6 (C-2, C-6), 126.8 (C-3′), 125.1 (C-2′), 123.3 (C-3, C-5), 118.4 (C-1′), 114.0 (C-4). LCMS (H+) calculated for C_12_H_13_N_3_O_2_S [M–H]^+^: 264.07, found: 264.09; anal. calculated (%): C, 54.74; H, 4.98; N, 15.96. Found: C, 54.69; H, 4.86; N, 15.80.

#### 2.3.10. (*E*)-N′-((1H-pyrrol-3-yl)methylene)-4-methoxybenzenesulfonohydrazide (**3j**)

Solid; reaction time, 12 h; yield, 65%; melting point: 176–179°C; *R*_f_ 0.41 (ethyl acetate : pet. ether 1 : 3), FTIR *n*_max_ cm^1^: 3291 (NH), 3093 (sp^2^ CH), 1626 (CN), 1473 (C=C aromatic). HPLC result of product: Daicel CHIRALPAK® AD-H column; 30% ethyl acetate in pet. ether; 1.0 mL/min; retention time: 29.3 min; 85.7% pure; ^1^H NMR (CDCl_3_, *d* ppm, *J* Hz); 7.77 (s, 1H, CH), 7.50 (*d*, *J* = 4.0, 2H, H-2, H-6), 7.44 (*d*, *J* = 4.0, 1H, H-3′), 7.25 (*d*, *J* = 4.0, 1H, H-4′), 7.22 (*d*, *J* = 4.0, 1H, H-5′), 7.17 (*d*, *J* = 4.0, 1H, H-6′), 7.14 (*d*, *J* = 4.0, 2H, H-3, H-5), 6.28 (s, 1H, NH), 2.31 (s, 1H, CH_3_). ^13^C NMR (CDCl_3_, *d* ppm); 145.5 (CH), 145.4 (C-1), 142.6 (C-1′), 138.5 (C-6′), 136.5 (C-4), 130.3 (C-5′), 128.6 (C-3, C-5), 127.6 (C-3′), 125.9 (C-2, C-6), 124.6 (C-4′), 21.7 (CH_3_). LCMS (H+) calculated for C_12_H_13_N_3_O_2_S [M–H]^+^: 280.31, found: 280.09; anal. calculated (%): C, 51.60; H, 4.69; N, 15.04. Found: C, 51.79; H, 4.86; N, 15.10.

### 2.4. Carbonic Anhydrase (CA) Assay

CA inhibition has been determined using some reported methods with a few modifications [23]. This method has been based on a principle which shows that para-nitrophenyl acetate upon hydrolysis by carbonic anhydrase produces yellow para-nitrophenol that has been measured using spectrophotometric technique. In brief, 120 microliters of 50 mM Tris-Sulfate buffer (with a pH 7.6 comprising 0.1 mM ZnCl_2_), twenty *μ*L of (50 U) bovine enzyme, and 20 *μ*L of inhibitor per well makes the reaction mixture. Mixture contents were mixed well before preincubation for ten minutes at 25°C. Para-nitrophenyl acetate substrate (6 mM stock solution by using <5% acetonitrile present in buffer and has been used fresh each time) and forty microliters were taken I each well to reach 0.6 mM concentration per well. The volume of the reaction mixture was diluted to 200 *μ*L. Contents were well mixed after thirty-minute incubation at 25°C, and absorbance results were determined at 348 nm with a microplate reader (SpectraMax ABS, USA). Standard used was acetazolamide. Experiments were performed in triplicate with each concentration. GraphPad Prism 5.0 (GraphPad, San Diego, CA, USA) has been used to calculate IC_50_ by nonlinear regression. Inhibition (%) = [(*B* − *S*)/*B*] × 100, where *S* and *B* are the absorbances of the samples and blank.

### 2.5. Kinetic Analysis

To determine the kinetic parameters of compound **3c**, a sequence of experiments have been done. Compound **3c** was selected on the basis of IC_50_ value which was used at different concentrations (0.0, 7.1, 14.2, and 28.4 *μ*M, respectively); the substrate (*p*-nitrophenyl acetate) concentrations were varied (2, 1, 0.5, 0.25, 0.125, and 0.0625 mM). Other all experimental conditions were just similar as described in the carbonic anhydrase assay segment. The maximum initial velocity has been measured using the starting linear part of absorbance values up to ten minutes at a 1-minute interval. Plots of Lineweaver–Burk on graph between inverse of velocities (1/*V*) and inverse of concentration of substrate 1/[*S*] mM^−1^ were used in order to find enzyme inhibition type. EI dissociation constant *K*_i_ was found by the secondary plot of concentrations of inhibitors versus 1/*V*.

### 2.6. Computational Methodology

#### 2.6.1. Retrieval of Carbonic Anhydrase in Maestro

The target protein/enzyme structure has been recovered using PDB (Protein Data Bank) (http://www.rcsb.org) having PDBID 1V9E. Structure of protein has been prepared using “Protein Preparation Wizard” workflow in Schrödinger Suite. The bond orders were allotted, and H atoms have been added with protein molecule. The water molecules were removed from protein structure. Then, structure was reduced to minimum to get the RMSD (converged root mean square deviation) of 0.30 Å with the OPLS_2005 force field. The prepared structure was employed for further grid and docking analysis.

#### 2.6.2. Grid Generation and Molecular Docking

For grid generation preparation, the enzymatic site of the target protein enzyme has been reached using cocrystallized ligands taken from PDB and the literature data. Grid was generated by specifying the particular residues involved in the active zone of the target protein. After grid preparation, docking experiment was performed against synthesized compounds **(3a-3l)** against receptor molecule. The synthesized ligands were sketched by 2D sketcher in Maestro interface and utilized in docking procedure. The default docking setup parameters were employed for ligand docking experiment. The binding energies and conformations/positions of ligands have been predicted within the region of activity of protein by glide experiment. The 3D and 2D graphical images of both best scored docking complexes were retrieved using Maestro.

#### 2.6.3. Cell Viability Assay Using HaCaT and MCF-7

The experiment for cell viability studies using MTT (3-(4,5-dimethylthiazol-2yl)-2,5-diphenyltetrazolium bromide) setup has been done to find cytotoxic effects of target compounds. The human keratinocyte (HaCaT) and MCF-7 were cultivated separately in 96-well plates (CGP, gamma sterilized, SPL Korea) with use of complete DMEM consisting of ten percent fetal bovine serum and one percent antibiotics. 5∗10^4^ cells per well were incubated for 24 hours before their exposure to a series of dilutions of target derivatives. The analogue **3c** was solubilized in DMSO, and DMEM was used for dilution to achieve final concentrations 0, 125, and 250 *μ*M. Then, in the presence of test compounds, cells were incubated again for twenty-four hours at 37 C with 5% carbon dioxide. Now, through the MTT setup, absorbance values at 570 nm were determined by ELISA reader and each experiment was repeated thrice. All data was shown in a mean ± standard deviation pattern, and Student's *t*-tests were performed to analyze significance of the data. Statistical significance has been assumed (*p* < 0.005).

## 3. Results

**Figure 1:**
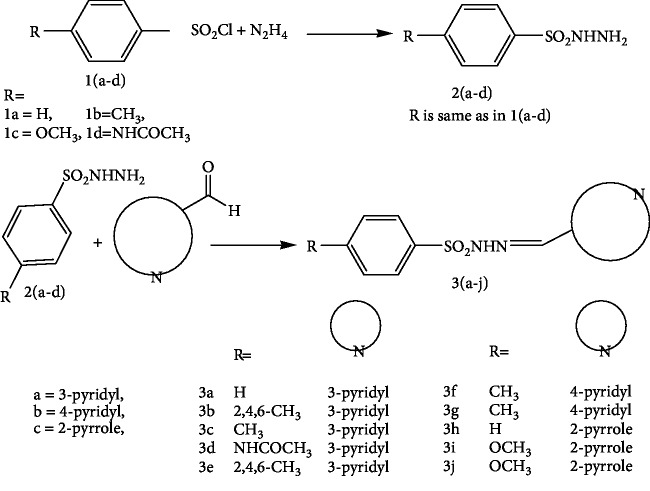
Lineweaver–Burk plots for inhibition of carbonic anhydrase with derivative **3c**. (a) Concentrations of **3c** were 0.00, 7.1, 14.2, and 28.4 *μ*M, respectively. Substrate *p*-nitrophenyl acetate concentrations were 0.0625, 0.125, 0.25, 0.5, 1, and 2 mM. (b) The inset shows the plot of the slope versus **3c** concentrations to find the inhibition constant. Lines were drawn by linear least squares fit.

**Figure 2:**
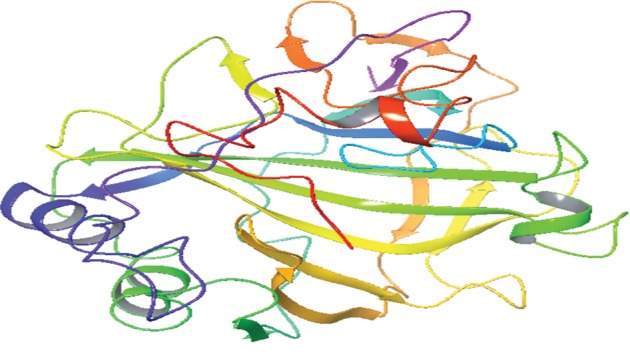
Crystal structure of bovine anhydrase II PDBID (1V9E).

**Figure 3:**
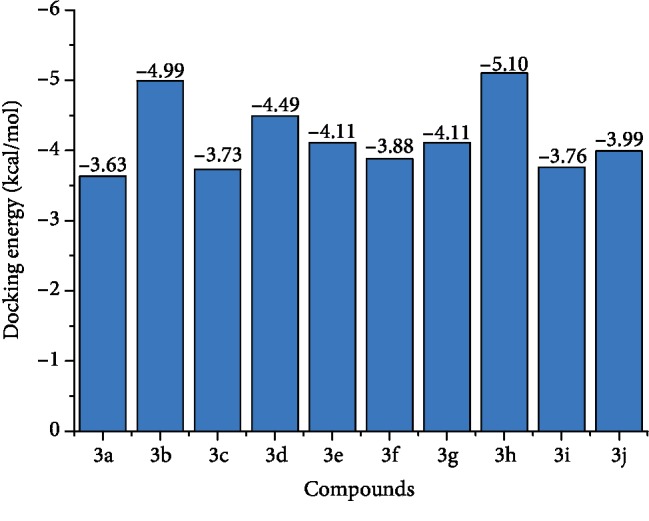
Glide docking score of the synthesized compounds **3a-j** docking complexes with PDBID (1V9E).

**Figure 4:**
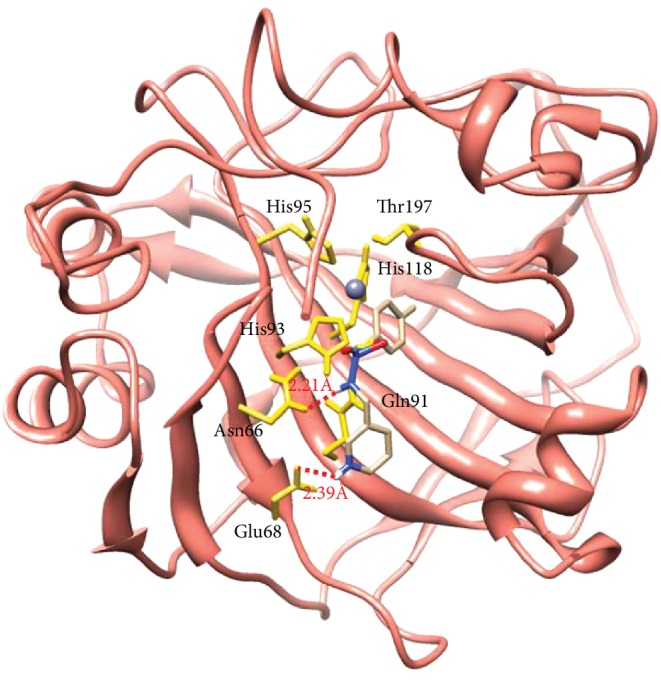
3D docking interactions of **3c** with target protein PDBID (1V9E).

**Figure 5:**
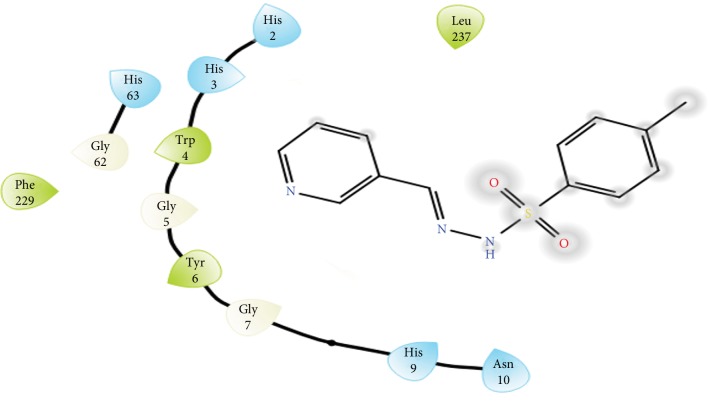
2D docking interactions of **3c** with target protein PDBID (1V9E).

The carbonic anhydrase inhibitory activity of the synthesized azaheterocyclic Schiff bases **(3a-j)** was performed, and results are presented in Table 1. The excellent activity was shown by compound **3c** with IC_50_0.84 ± 0 s.12 *μ*M value better than the standard acetazolamide (IC_50_0.99 ± 0.04 *μ*M). Kinetic results of CA were determined by Lineweaver-Burk plots of 1/*V* versus 1/[S] using different concentrations of target analogues. They produced a set of straight line (Figure 1(a)). To investigate the mechanism of inhibition of target analogues on carbonic anhydrase inhibition, a kinetic study was done. Depending on the best IC_50_ value, compound **3c** was selected to find out the mechanism of an enzyme inhibition. Kinetics of analogue **3c** expressed that it intersects within the second quadrant. The analysis expressed that *V*_max_ decreased by increasing concentrations of inhibitors while *K*_m_ remains same. The Ramachandran plots showed that 93.8% of all remainder was existing in preferred regions (Figure 2). Virtual screening revealed that glide docking energy values were little fluctuated among all ligands **(3a-3j)** and exhibited well docking energy values, respectively (Figure 3). The comparative results showed that docking energy values of target derivatives were closer to each other. The ligands-protein binding analyses expressed **3c** confined in the active site of the target protein as shown in Figures 4 and 5. The **3c** receptor complex revealed a good conformation with a better interaction style in the binding pocket of the receptor. Result of **3c** receptor docked complex revealed that hydrogen bonding and hydrophobic interactions were found at Asn66 and Glu68, respectively.

**Figure 6:**
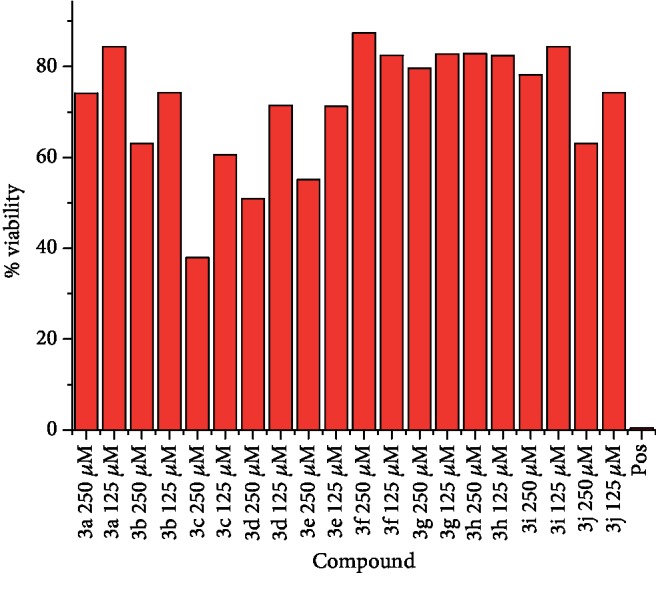
The effects of azaheterocyclic sulfonamide Schiff bases on cell viability of the MCF-7 cells.

**Figure 7:**
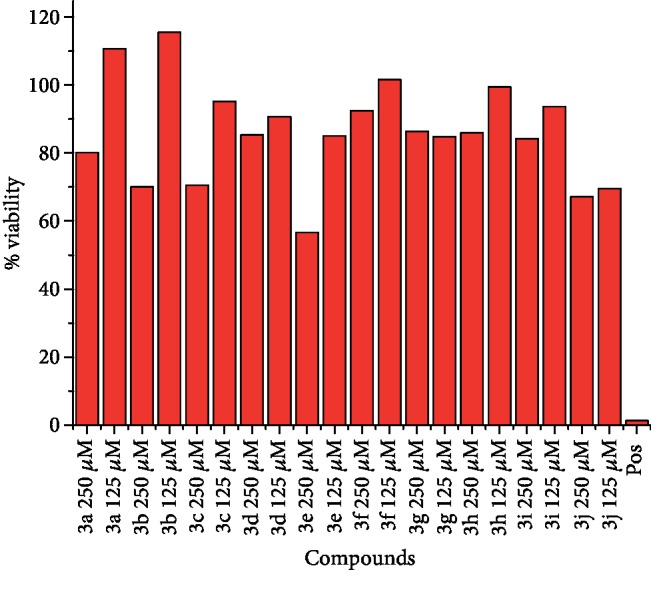
The effects of azaheterocyclic sulfonamide Schiff bases on cell viability of the human keratinocyte (HaCaT) cells.

Furthermore, the present study summarized that derivative **3c** caused cytotoxicity in a concentration-dependent manner as shown in Figures 6 and 7. The cytotoxic effects produced by derivative **3c** are smaller in case of human keratinocyte (HaCaT) at concentration 250 *μ*M than MCF-7.

## 4. Discussion

The *p*-substituted benzene sulphonyl hydrazides **2(a-d)** were obtained by condensation of *p*-substituted benzene sulphonyl chloride with hydrazine. The presence of -NH stretching at 3304 cm^−1^ and 3107 cm^−1^ in FTIR spectra showed the successful synthesis of intermediate hydrazides. Intermediate hydrazides **2(a-d)** were condensed with *p*-substituted benzaldehydes to reach target compounds **3(a-j)**. All the target derivatives were characterized using FTIR, ^1^H NMR, and ^13^C NMR. The position and type of substitution in the synthesized compounds are the determinant factor of enzyme inhibitory activity. It was observed that derivatives with the pyrrole ring are less active than those which have the pyridine ring. The electron density at the heterocyclic pyridine ring is lesser compared to the five-member pyrrole ring. The presence of the pyridyl ring in compound **3c** along with the presence of the para-methyl-substituted phenyl ring plays a vital role in carbonic anhydrase inhibitory activity. The derivative **3c** inhibits the CA II noncompetitively to give rise to the enzyme inhibitor complex. The secondary plot of the slope against the doses of inhibitors expressed the enzyme inhibitor dissociation constant (*K*_i_) (Figure 1(b)). The kinetic parameters are summarized in Table 2 which shows the noncompetitive mode of inhibition. Carbonic anhydrase II is a (Zn) metal-comprising protein and consists of 259 amino acids. Residual structure of CA II contains 9% helices, 45% *β* sheets, and 45% coils. The Ramachandran graph and results confirmed the consistency and effectiveness of CA II structure. The comparative results of the docking study enunciated that docking energy values of target derivatives were closer to each other as their basic skeleton was similar in all synthesized compounds. All the compounds were docked against target protein PDBID (1V9E). Based on in vitro results, the **3c** docking complex was selected to determine their binding pattern and conformational position in the active site of the target protein (**3c** confined in the active site of the target protein). The hydrogen atom of the amino group forms a hydrogen bond with His3, whereas the benzene ring forms a partially weak hydrophobic interaction with Trp4 having bond lengths 2.11 and 2.39 Å, respectively. Literature data also favor our docking results, and similar residues were involved in different inhibitors [24–26]. The two-dimensional conformations of each docking complex are mentioned in supplementary data (Figures [Supplementary-material supplementary-material-1]). Based on our findings, the azaheterocyclic Schiff base **3c** expressed the most potent carbonic anhydrase inhibition and high binding affinity with carbonic anhydrase enzyme. Viability assay of these derivatives on human keratinocyte (HaCaT) and MCF was performed (Figures 6 and 7).

## 5. Conclusion

The azaheterocyclic Schiff base derivatives with various hydrophobic or hydrophilic groups have been synthesized to explore their potential in carbonic anhydrase inhibition. The derivative **3c** represented comparable carbonic anhydrase inhibition (IC_50_ 0.84 *μ*M) with standard acetazolamide (IC_50_ 0.99 *μ*M). The kinetic investigations expressed that derivative **3c** exhibited a mixed-type inhibition of carbonic anhydrase enzyme. It expressed less cytotoxic effects on human keratinocyte cell lines (HaCaT) compared to breast cancer cell lines MCF-7. The computational studies also assured that derivative **3c** binds well in the active binding site of the target enzyme. The highest binding affinity of **3c** confirmed that the methyl group at the phenyl ring and the sulfonamide group in compound **3c** play an important role in giving stable ligand–target complex as well as in carbonic anhydrase inhibition.

## Figures and Tables

**Table 1:** Carbonic anhydrase inhibitory activity of azaheterocyclic Schiff bases **3a-j**.

Compound	Carbonic anhydrase IC_50_ ± SEM (*μ*M)
3a	No inhibition
3b	No inhibition
3c	0.84 ± 0.12
3d	No inhibition
3e	18.86 ± 0.85
3f	No inhibition
3g	59.12 ± 1.68
3h	31.75 ± 1.07
3i	No inhibition
3j	No inhibition
Acetazolamide	0.99 ± 0.04

**Table 2:** Kinetic parameters of the compound **3c** on carbonic anhydrase.

Concentration (*μ*M)	*V*_max_ (*Δ*A/min)	*K*_m_ (mM)	Inhibition type	*K*_i_ (*μ*M)
0.00	0.016505745	0.42	Noncompetitive	8.6
7.1	0.010063181	0.42		
14.2	0.004551758	0.42		
28.4	0.002292984	0.42		

*K*_m_ is the Michaelis-Menten constant, *V*_max_ is the reaction velocity, and *K*_i_ is the EI dissociation constant.

## Data Availability

Present work was a part of Ph.D thesis of a research scholar Mujahid Abas, supervised by Dr. Zaman Ashraf generated at Allama Iqbal Open University, Islamabad and College of Natural Sciences, Kongju National University, Gongju, Korea. Dr. Zaman Ashraf may provide data upon request.

## Supplementary Materials

Supplementary Materials Figure S1: docking interaction 3a with target protein PDBID (1V9E). Figure S2: docking interaction 3b with target protein PDBID (1V9E). Figure S3: docking interaction 3c with target protein PDBID (1V9E). Figure S4: docking interaction 3d with target protein PDBID (1V9E). Figure S5: docking interaction 3e with target protein PDBID (1V9E). Figure S6: docking interaction 3f with target protein PDBID (1V9E). Figure S7: docking interaction 3g with target protein PDBID (1V9E). Figure S8: docking interaction 3h with target protein PDBID (1V9E). Figure S9: docking interaction 3i with target protein PDBID (1V9E). Figure S10: docking interaction 3d with target protein PDBID (1V9E).
