# Supplementary material for: Sulfonamide-Based Azaheterocyclic Schiff Base Derivatives as Potential Carbonic Anhydrase Inhibitors: Synthesis, Cytotoxicity, and Enzyme Inhibitory Kinetics
Source: Biomed Res Int. 2020 Feb 20;2020:8104107. doi: 10.1155/2020/8104107 (PMC7054763; doi:10.1155/2020/8104107)
Supplement: Supplementary Materials — Figure S1: docking interaction 3a with target protein PDBID (1V9E). Figure S2: docking interaction 3b with target protein PDBID (1V9E). Figure S3: docking interaction 3c with target protein PDBID (1V9E). Figure S4: docking interaction 3d with target protein PDBID (1V9E). Figure S5: docking interaction 3e with target protein PDBID (1V9E). Figure S6: docking interaction 3f with target protein PDBID (1V9E). Figure S7: docking interaction 3g with target protein PDBID (1V9E). Figure S8: docking interaction 3h with target protein PDBID (1V9E). Figure S9: docking interaction 3i with target protein PDBID (1V9E). Figure S10: docking interaction 3d with target protein PDBID (1V9E). [file 8104107.f1.docx]

**Supplementary File**

**Sulfonamide Based Aza-Heterocyclic Schiff Bases Derivatives as a Potential Carbonic Anhydrase Inhibitors: Synthesis, Cytotoxicity and Enzyme Inhibitory Kinetics**

**Mujahid Abas^1^, Hummera Rafique^2^, Shazia Shamas^3^, Sadia Roshan^3^, Zaman Ashraf^1^, Zafar Iqbal^1^, Hussain Raza^4^, Mubashir Hassan^5^, Khurram Afzal^6^, Albert A Rizvanov^7^, and Muhammad Hassham Hassan Bin Asad^7, 8^**

*^1^ Department of Chemistry, Allama Iqbal Open University, Islamabad 44000, Pakistan.*

*^2^ Department of Chemistry, University of Gujrat, Gujrat-50700, Pakistan*

*^3^ Department of Zoology, University of Gujrat, Gujrat-50700, Pakistan*

*^4^ Department of Biological Sciences, College of Natural Sciences, Kongju National University, Gongju, 314-701, Korea*

*^5^ Institute of Molecular Biology and biotechnology, The University of Lahore, Pakistan.*

*^6^ Institute of Food Sciences, Bahauddin Zakria University, Multan 60800, Pakistan*

*^7^Institute of Fundamental Medicine and Biology, Department of Genetics, Kazan Federal University, 420008, Kazan, Russia*

*^8^Department of Pharmacy, COMSATS University Islamabad, Abbottabad Campus 22060, Pakistan*

Correspondence should be addressed to Dr. Zaman Ashraf; [mzchem@yahoo.com](mailto:mzchem@yahoo.com) and Dr. Muhammad Hassham Hassan Bin Asad; [hasshamasad@yahoo.com](mailto:hasshamasad@yahoo.com)

**
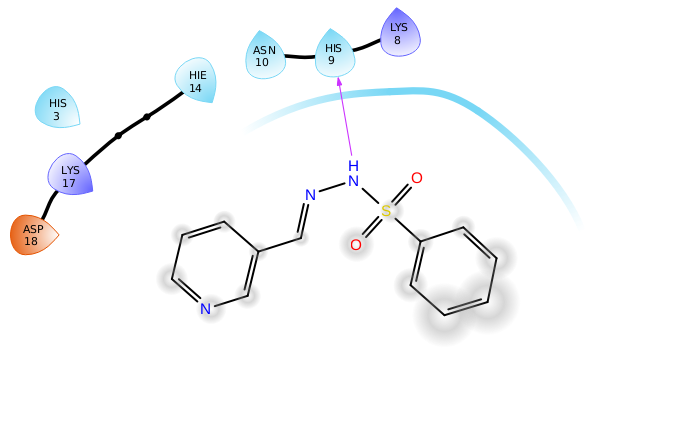
**

F_IGURE_ S1: Docking interaction 3a with target protein PDBID (1V9E).


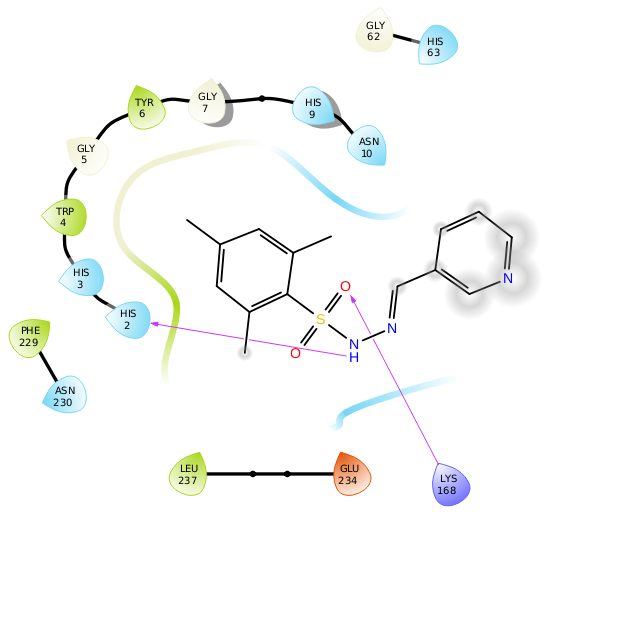


F_IGURE_ S_2_: Docking interaction 3b with target protein PDBID (1V9E).


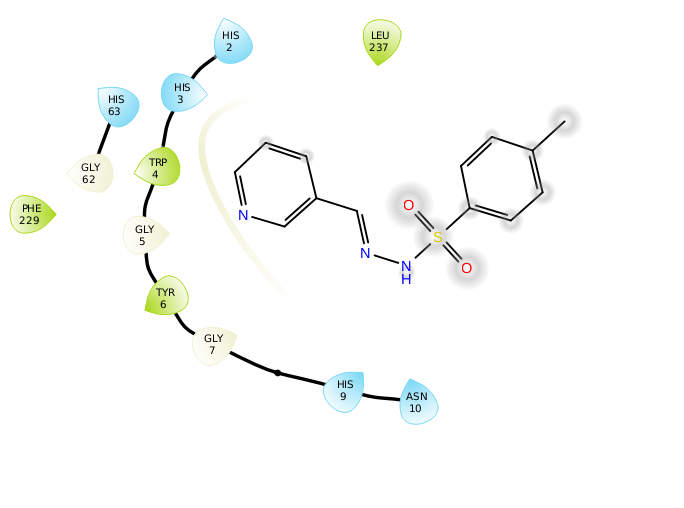


F_IGURE_ S_3_: Docking interaction 3c with target protein PDBID (1V9E).


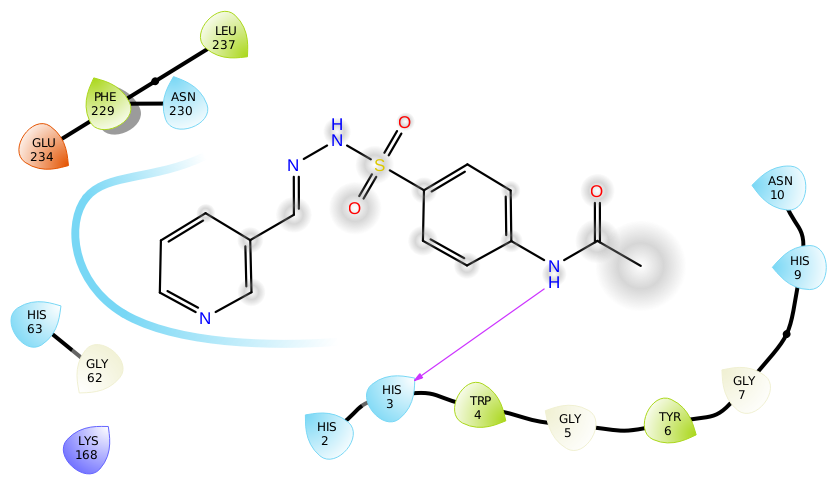


F_IGURE_ S_4_: Docking interaction 3d with target protein PDBID (1V9E).


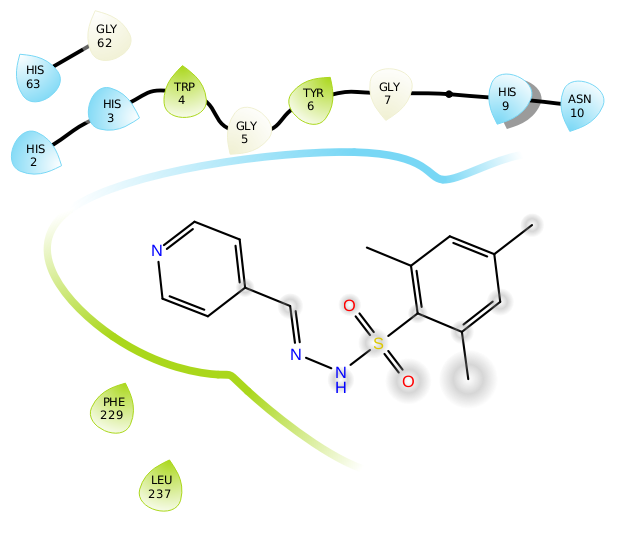


F_IGURE_ S_5_: Docking interaction 3e with target protein PDBID (1V9E).


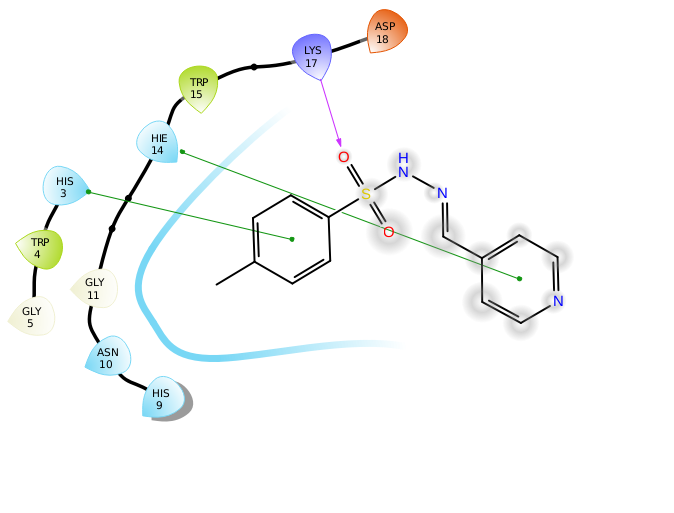


F_IGURE_ S_6_: Docking interaction 3f with target protein PDBID (1V9E).


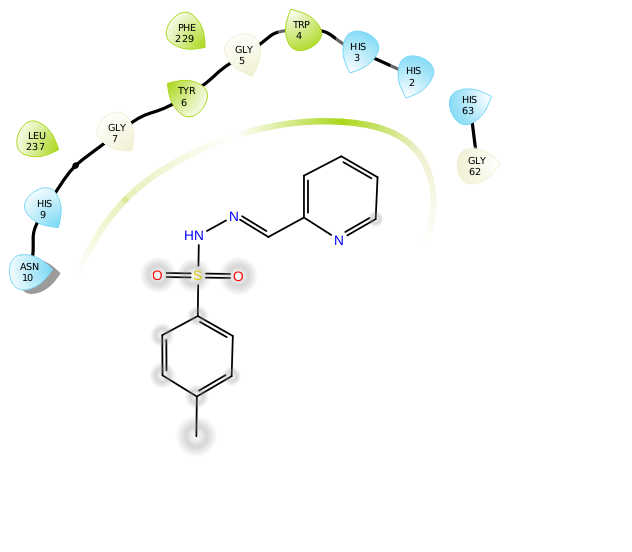


F_IGURE_ S_7_: Docking interaction 3*g* with *target protein PDBID (1V9E).*


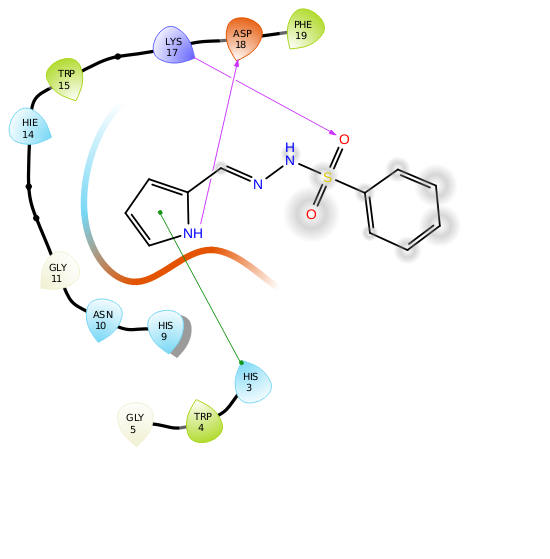


F_IGURE_ S_8_: Docking interaction 3h with target protein PDBID (1V9E).


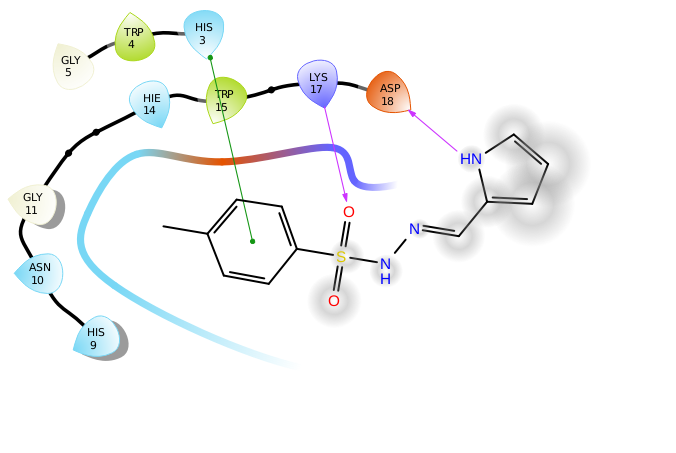


F_IGURE_ S_9_: Docking interaction 3i with target protein PDBID (1V9E).


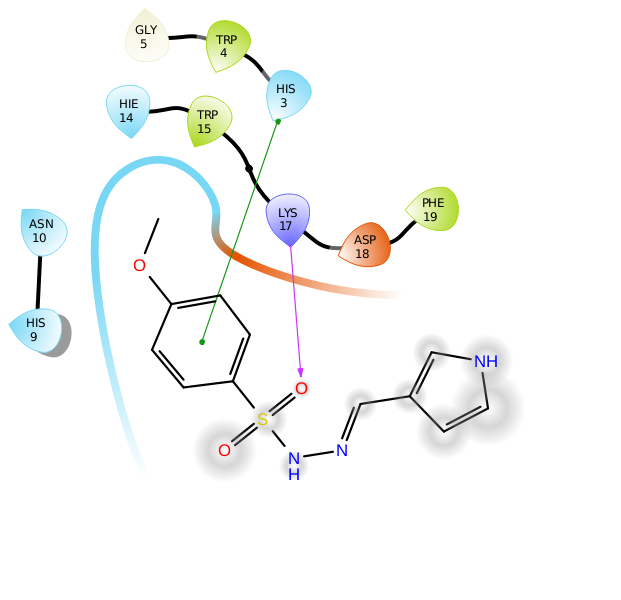


F_IGURE_ S_10_: Docking interaction 3d with target protein PDBID (1V9E).
